# Developing an International Combined Applied Surgical Science and Wet Lab Simulation Course as an Undergraduate Teaching Model

**DOI:** 10.1155/2015/463987

**Published:** 2015-11-03

**Authors:** Michail Sideris, Apostolos Papalois, Georgios Tsoulfas, Sanjib Majumder, Konstantinos Toutouzas, Efstratios Koletsis, Panagiotis Dedeilias, Nikolaos Lymperopoulos, Savvas Papagrigoriadis, Vassilios Papalois, Georgios Zografos

**Affiliations:** ^1^King's College Hospital NHS Foundation Trust, Denmark Hill, London SE5 9RS, UK; ^2^Experimental Research Centre of ELPEN, 19009 Pikermi, Greece; ^3^Aristotle University of Thessaloniki, 54124 Thessaloniki, Greece; ^4^Pinderfields Hospital, NHS, Wakefield, West Yorkshire WF1 4DG, UK; ^5^University of Leeds, Leeds LS2 9JT, UK; ^6^University of Athens, Goudi, 11527 Athens, Greece; ^7^University of Patras, 26500 Rio, Greece; ^8^Evangelismos Hospital, 10676 Athens, Greece; ^9^King's College London, Strand WC2R 2LS, UK; ^10^Hammersmith Hospital, London W12 0HS, UK; ^11^Imperial College London, London SW7 2AZ, UK; ^12^Hippocration University Hospital, 11528 Athens, Greece

## Abstract

*Background*. Essential Skills in the Management of Surgical Cases (ESMSC) is an international, animal model-based course. It combines interactive lectures with basic ex vivo stations and more advanced wet lab modules, that is, in vivo dissections and Heart Transplant Surgery on a swine model. *Materials and Methods*. Forty-nine medical students (male, *N* = 27, female *N* = 22, and mean age = 23.7 years) from King's College London (KCL) and Greek Medical Schools attended the course. Participants were assessed with Direct Observation of Procedural Skills (DOPS), as well as Multiple Choice Questions (MCQs). Paired *t*-test associations were used to evaluate whether there was statistically significant improvement in their performance. *Aim*. To evaluate the effectiveness of a combined applied surgical science and wet lab simulation course as a teaching model for surgical skills at the undergraduate level. *Results*. The mean MCQ score was improved by 2.33/32 (*P* < 0.005). Surgical skills competences, as defined by DOPS scores, were improved in a statically significant manner (*P* < 0.005 for all paired *t*-test correlations). *Conclusions*. ESMSC seems to be an effective teaching model, which improves the understanding of the surgical approach and the basic surgical skills. In vivo models could be used potentially as a step further in the Undergraduate Surgical Education.

## 1. Introduction

Animal model-based simulation has been used for training purposes throughout the time. Most of the current wet lab simulation models have been used for advanced postgraduate training [1–6]. The overall outcome of these courses seems to be satisfactory [2, 3]. Despite the fact that most of these courses reflect advanced training, recently there are some emerging new ones that may be appropriate for undergraduates [7, 8].

ESMSC is a two-day international course, which combines applied surgical science lectures with wet lab in vivo and ex vivo simulation skills' modules on a swine model. The course curriculum has been organized effectively in three main cores. The first core refers to four basic science workshops (BScCI), which contain interactive teaching, on the principles of shock and fundamental interpretation of arterial blood gases (ABGs), electrocardiography (ECG), as well as principles in the management of fluids and analgesia. The second core includes case-based interactive lectures (CbCII) on main surgical specialties, which aim to familiarize the participants with a common pattern of approach. This refers to a safety pyramid (Figure 6), which implies a systematic approach for every surgical case. The third core refers to the in vivo and ex vivo wet lab simulation modules (SkCIII), which occupy 50% of the course curriculum. The novelty of our model lies in the combination of basic knowledge that is required to deal with a patient, that is, fluid resuscitation, ECG, Acid-Base Balance (Basic Science Core), with the principles of a systematic, methodical and safe approach of every surgical patient (Case-Based Lectures and safety pyramid, Figure 6), and the acquisition of fundamental skills required for many common procedures, that is, suturing, IV access, Wound Debridement, chest drain insertion, and basic dissections (Skills' Core). The second unique part of our curriculum is the exposure in some promising in vivo experience, which hopefully drives students' initiative and at the same time expands their ability to effectively perform basic procedures and assist in theatre. Also, watching step by step a live heart transplant would be the highest quality teaching towards the understanding and the consolidation of cardiopulmonary physiology principles. Despite being deemed as an advanced module, it still maintains its teaching and mentorship value even for medical students. The overall idea is to create a curriculum, which will serve as a preparatory step for delivering a generation of well-motivated and efficient future junior trainees in surgery.

In the first in vivo model, participants are taught how to insert a chest tube, demonstrate basic chest anatomy, dissect the abdominal wall, repair primarily perforated bowel tissue, perform a Diagnostic Peritoneal Lavage (DPL), and achieve haemostasis on an actual liver laceration. The second in vivo experiment uses a second anaesthetized pig, in order to demonstrate the principles of extracorporeal circulation. Participants actively assist an experienced team of senior surgeons and enjoy real time teaching on the fundamentals of Heart and Transplant Surgery.

Ex vivo stations include a basic suturing station, where participants are instructed by a team of Plastic Surgeons (SpR and above) on how to perform (and aided in doing so) interrupted, subcuticular, and mattress sutures on swine skin flaps. A balloon is supporting the flap on top of a box, simulating the peritoneum. Another station includes intravenous (IV) access skills and the Seldinger technique for central lines. The third ex vivo station refers to Wound Debridement of swine flaps and primary closure of lacerations. Finally, the last station consists of Open Reduction Internal Fixation (ORIF) of hand fractures, where participants have the chance to familiarize themselves in the relevant technique.

For the purposes of the course, a detailed manual has been published (Scientific Publications Parisianou, ISBN: 978-960-583-063-2), containing 30 chapters from King's College in London and Greek Universities.

## 2. Materials and Methods

**Figure 1:**
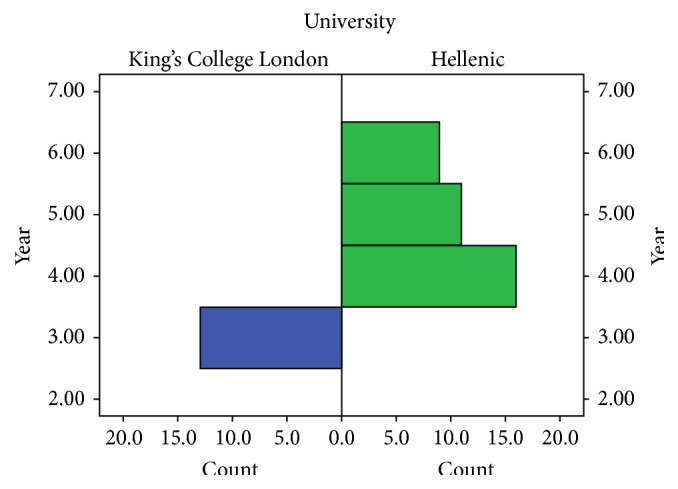
Distribution of participants in year of studies.

Forty-nine delegates (male *N* = 27, female *N* = 22) were selected and assessed throughout the course. The selection criteria were based on CV parameters including previous oral or poster presentations and publications. The whole process ran through the online portal (http://esmsc.gr/), and the applicants were also asked to write a small statement explaining the reasons for their application, in order to assess their personal interest in surgical specialties. The mean age was 23.7 years (min = 20, max = 30, and SD = 2.47). Out of these, 26.5% (*N* = 13) were students from KCL at Year 3 and the remaining 73.5% (*N* = 36) from Greek Universities, from which 32.7% were Year 4 (*N* = 16), 22.4% Year 5 (*N* = 11), and 18.4% were Year 6 (*N* = 9) (Figure 1). KCL Year 3 is the first clinical rotation of the students and the experience is equal to 4th and 5th Year in Greek Universities. None of the medical students had any previous experience in wet lab courses or the operating room, and therefore their relevant experience was assumed to be similar.

The course manual (2nd Edition) was designed to cover all the background knowledge that is required for the consolidation of the course curriculum, and it was given to the students, on their arrival.

Precourse MCQ exam was performed on participants' arrival, and the same exam was conducted on completion of the course. This MCQ reflects on the course curriculum and consists of 32 questions of average difficulty. Participants were assessed before and after every wet lab skills' module, with DOPS assessments, by qualified trainers from the UK and Greece. All DOPS forms were validated by the ISCP (Table 1), simplified and standardized for the purposes of the course. We used 3 different markings, N (or 0) for not able to perform (or not observed), D (or 1) for development needed, and S (or 2) for satisfactory. This reflects on specific parts of each wet lab module. Global marking on scale 0–4 was used to assess the overall competence of the delegates to perform each module independently or with assistance.

**Figure 2:**
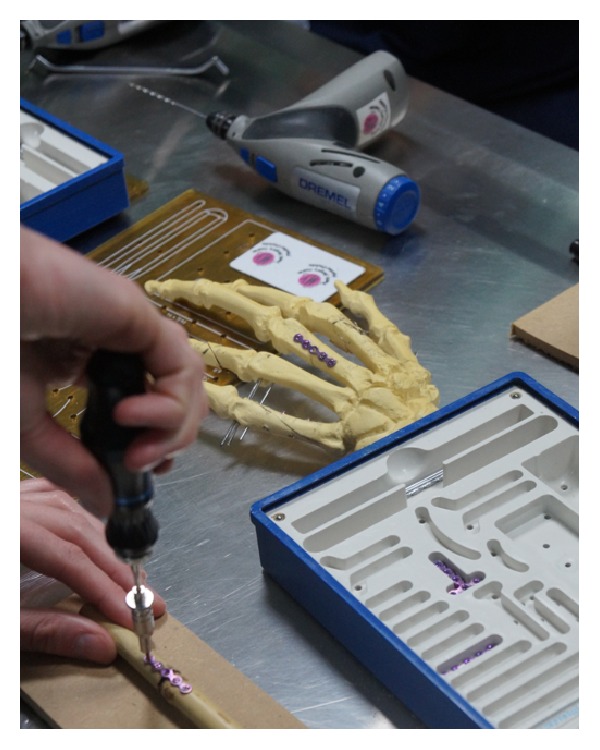
Open Reduction Internal Fixation of hand fractures.

**Figure 3:**
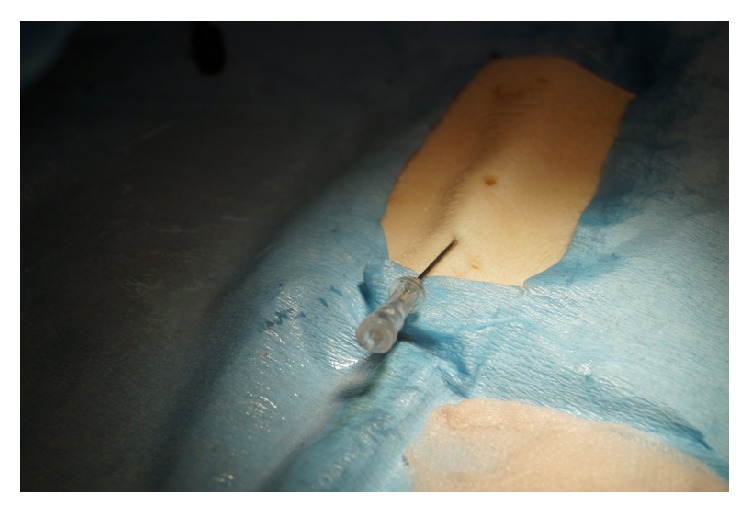
IV Seldinger technique using pig skin flaps.

On the suturing station, participants were assessed on the ability to perform independently interrupted, subcuticular, and mattress sutures (Figure 5). During the Wound Debridement module, delegates were assessed on their ability to clean a wound and suture it primarily. Using certain questions, they were also prompted on their understanding of the process. ORIF station delegates were expected to perform the relevant skill on small plastic pieces, using the relevant equipment (Figure 2). The IV Seldinger technique was conducted (Figure 3) on swine skin flaps, using urethral catheters and the relevant equipment. Participants were tested on their ability to understand the indications and the complications and independently perform the procedure.

**Figure 4:**
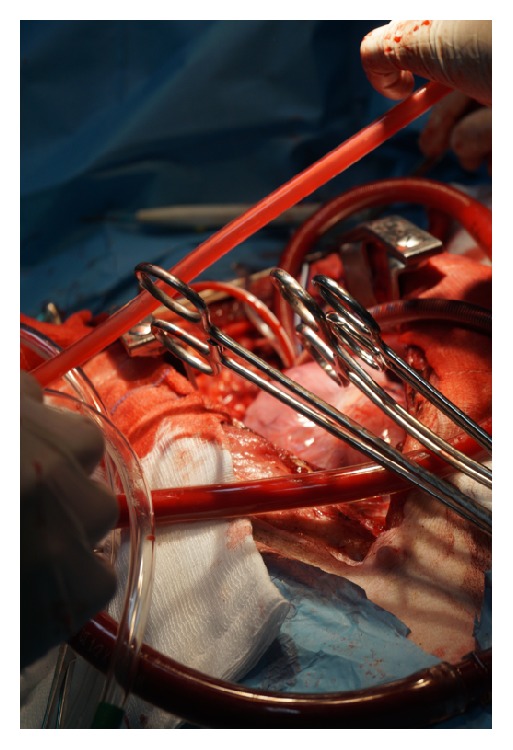
Heart and Transplant In Vivo Module on swine model.

**Figure 5:**
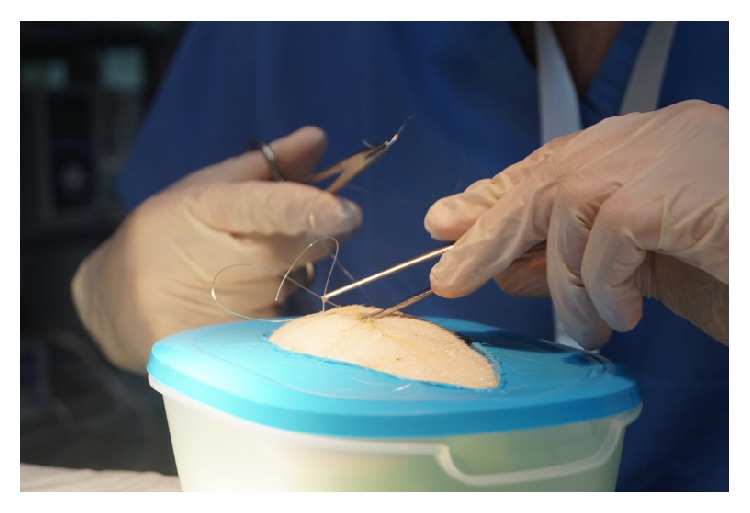
Suturing station on pig skin flaps.

**Figure 6:**
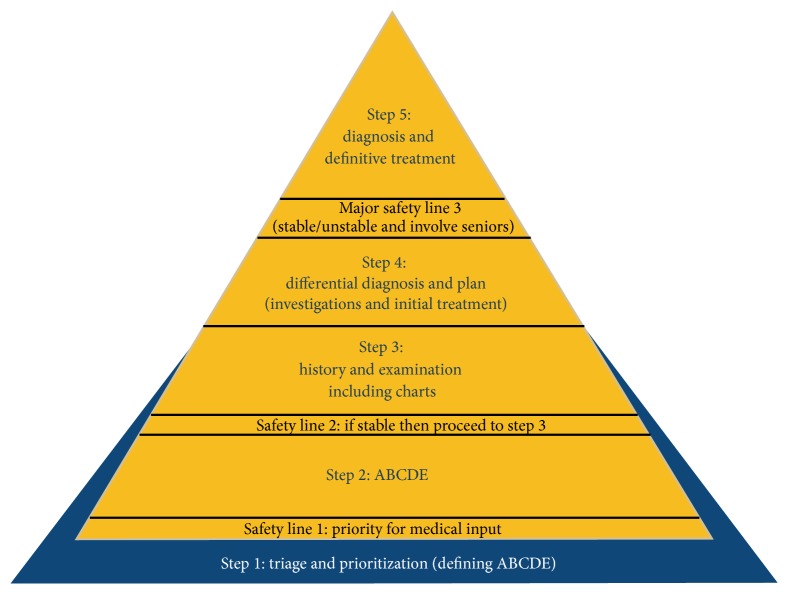
Safety triangle as published on the relevant manual (Sideris, Papalois et al.).

With regard to the in vivo experiments, local standard operational procedure (SOP) protocol for anaesthesia and preparation of the pig was used accordingly, whilst on the first in vivo experiment participants were actively assisting on anatomy demonstrations, as well as chest tube insertion. They were tested on their ability to perform abdominal wall dissection through layers independently and closure. DOPS assessments were used on the same basis as before. During the second in vivo experiment, delegates had the opportunity to assist a senior surgeon preparing the pig for heart transplant (Figure 4). During this module, we used only the global rating scale (0–4), as it was above the level of expected skills.

Detailed feedback forms were handled to the delegates on their arrival. A global rating scale of 1–10 was used for every course lecture or skills module. Overall satisfaction questions were included on it in order to acquire an idea of the overall setting of the course.

Statistical analysis of the MCQ and DOPS results was conducted using* paired t-test associations* before* and after each module.* Independent *t*-test associations were performed to assess the difference in the performance between different groups of participants, that is, Years 3 and 4 versus Years 5 and 6 students, as well as UK versus Greek students. For this purpose, we used IBM SPSS for Mac (Edition 22, Armonk, NY: IBM Corp.). *P* values less than .05 were thought to be statistically significant.

## 3. Results

Mean MCQ score of the delegates before the course was 15.32/32 (min = 9, max = 22, and SD = 3.63) versus 18.2/32 (min = 9, max = 25, and SD = 3.93) after course. The mean difference was 2.67 (min = 1.51, max = 3.81, and SD = 3.23, *P* < 0.005). With regard to wet lab skills' module, the mean score of in vivo dissections (Skill 1) before teaching was 0.23 out of 2 (min = 0, max = 1, and SD = 0.43) with a mean global rating (scale 0–4) of 1.2 (min = 1, max = 2, and SD = 0.41), versus postteaching mean = 1.21 out of 2 (min = 1, max = 2, and SD = 0.41) and mean global rating of 2.5 (min = 2, max = 3, and SD = 0.51, score 0–4). The mean difference before and after in vivo dissections teaching was 0.96 out of 2 (min = 0.80, max = 1.10, and SD = 0.36, *P* < 0.005) and the mean global rating improvement was 1.29 (min = 0.93, max = 1.63, and SD = 0.61, *P* < 0.005) (Tables 2 and 3).

With regard to ex vivo suturing station (Skill 2), the mean score before teaching was 0.82 out of 2 (min = 0, max = 1, and SD = 0.39) with a global rating of 1.94 (min = 1, max = 3, and SD = 0.44, scale 0–4). The mean score after teaching was 1.90 out of 2 (min = 1, max = 2, and SD = 0.30) and the mean global rating was 2.94 (min = 2, max = 4, and SD = 0.44). The mean difference before and after teaching was 1.09 (min = 0.96, max = 1.23, and SD = 0.30, *P* < 0.005) with a global rating improvement of 1.00 (min = 0.72, max = 1.27, and SD = 0.52, *P* < 0.005) (Tables 2 and 3).

Delegates mean score in the IV Access Ex Vivo station (Skill 3) before teaching was 0.76 out of 2 (min = 0, max = 2) with mean global rating of 1.42 (min = 1, max = 3, and SD = 0.64, scale 0–4). The mean score after teaching was 1.66 out of 2 (min = 1, max = 2, and SD = 0.48) with a mean global rating of 2.35 (min = 2, max = 4, and SD = 0.63, scale 0–4). The mean difference in the score was 0.90 (min = 0.76, max = 1.04, and SD = 0.30, *P* < 0.005) and in the global rating was 0.92 (min = 0.77, max = 1.08, and SD = 0.26) (Tables 2 and 3).

The mean score in the ORIF ex vivo station (Skill 4) before teaching was 0.83 out of 2 (min = 0, max = 1, and SD = 0.38) and the mean global rating was 1.91 (min = 0, max = 3, and SD = 0.66, scale 0–4). The mean score after teaching was 1.88 out of 2 (min = 1, max = 2, and SD = 0.32) and the mean global rating was 3.08 (min = 2, max = 4, and SD = 0.51). The mean difference in the score was 1.05 (min = 0.85, max = 1.26, and SD = 0.41, *P* < 0.005) and in the global rating was 1.16 (min = 0.91, max = 1.41, and SD = 0.39, *P* < 0.005) (Tables 2 and 3).

In the Wound Debridement ex vivo module (Skill 5), the mean score before teaching was 1.05 out of 2 (min = 0, max = 2, and SD = 0.42) and the mean global rating was 1.61 (min = 0, max = 2, and SD = 0.65, scale 0–4). The mean score after teaching was 2.00 out of 2 (min = 2, max = 3, and SD = 0.00) and the mean global rating was 2.84 (min = 2, max = 3, and SD = 0.38, scale 0–4). The mean difference in the score was 0.93 (min = 0.7, max = 1.17, *P* < 0.005) and in the Global Score was 1.23 (min = 0.87, max = 1.59, and SD = 0.59, *P* < 0.005).

Global rating of the delegates for the Heart and Transplant In vivo Module (Skill 6) before teaching was 0.75 (min = 0, max = 1, and SD = 0.50, scale 0–4) versus mean global rating of 1.75 (min = 1, max = 2, and SD = 0.5, scale 0–4). The mean difference was 1.10 (min = 0.83, max = 1.32, and SD = 0.31, *P* < 0.005) (Tables 2 and 3).

With regard to independent *t*-test associations, the mean MCQ score after the course was 15.92 for UK students (Year 3) versus 19.29 for Greek Students (Year 4–6). There was a statistically significant better performance of Greek Students in the mean MCQ score after the course (*P* = 0.09, *P* = 0.05). The mean score in the in vivo dissections before and after completion of module (Skill 1) was .00 and 1.00 for the UK (Year 3) students versus 0.38 and 1.36 for (Year 4–6) Greek students. Therefore, there was an improved performance of Greek Students in the in vivo dissections' score before (*P* = 0.027, *P* = 0.09) and after the completion of module (*P* = 0.034, *P* = 0.019). There was no other statistical significant difference in any comparison between UK and Greek students.

Concerning the comparison between the 2 groups of Year 3 and Year 4 students versus Year 5 and Year 6 students, there was a statistically significantly better performance of the group of Year 5 and Year 6 students in the mean MCQ score after completion of course (mean score 20.37 versus 16.77, *P* = 0.034 and *P* = 0.014) as well as in the mean score before starting the in vivo dissections' module (Skill 1) (0.55 versus 0.06, *P* = 0.03 and *P* = 0.023). There was no other statistically significant difference in the performance of those 2 groups.

The mean overall feedback from the participants was 8.78 out of 10 (min = 7.72, max = 9.87, and SD = 0.56). There are those who think that ESMSC should be provided by the Medical School (9.86 out of 10, min = 8, max = 10, and SD = 0.43). Also, the overall idea was ranked with 9.42 out of 10 (min = 8, max = 10, and SD = 0.71) and the general concept of the curriculum was given 9.34/10 (min = 7, max = 10, and SD = 0.82). On direct questioning, the delegates think that this course helps in developing the principles of surgical approach (9.5 out of 10, min = 7, max = 10, and SD = 0.86) and the curriculum was given 9.30/10 in terms of how useful this may be for their future surgical career (min = 6, max = 10, and SD = 0.99). Most of the attendees seem to be interested in a surgical career (mean = 8.18, mean = 3, max = 10, and SD = 1.97). Detailed feedback is summarized in Table 4.

## 4. Discussion

Quality of surgical training is one of the biggest challenges that reflect directly onto patients' safety. There have been multiple models which aim to teach either basic [7, 8] or advanced skills [1, 2, 4–6] throughout the surgical journey. Surgical training is changing throughout Europe as the result of new legislation for working hours. Increasing workload along with the European Working Time Directive (EWTD) could potentially result in shorter surgical specialty training, which many have an impact on the adequacy of the training years in specialty [9]. This creates an argument for starting surgery-orientated training and skills-based training while still in Medical School.

On the other hand, increasing financial pressure is an additional challenge that has to be dealt with [10, 11]. Recently, bench model-based teaching of clinical skills has gained wide acceptance, though the outcomes still depend on the quality of facilities and trainers [10]. The need of getting trainees exposed to a wider variety of surgical skills before assisting in the theatre room is all the more clear given the increasing workload. Practicing skills in theatre could even double up the operating time [11] and even in some cases compromise patients' safety.

What is more, there has been a revolution of new surgical technologies in the last 15 years, such as laparoscopic surgery, robotic surgery, surgical endoscopy, and Natural Orifice Endoluminal Surgery. All those technologies have a steep learning curve and require long hands-on training. All surgical training tends to increasingly involve simulation training, which has been copied from the airline industry. This way of training is likely to develop into the major pattern of training for the current medical students.

There is no doubt that developing simulation models on which trainees can practice their skills would be beneficial for their long-term acquisition of required competences, in order to become safe and efficient surgeons [12, 13]. The main question though remains at which stage the right time to introduce future surgeons in those courses is. Moreover, it is vital to think about the right choice of each course curriculum, with specific aims and targets [14, 15], and subsequently to establish an objective validation model, which would prove that the outcome is worth money, time, and effort. There have been multiple validation methods [13], and in our case we used the Workplace Based Assessments (WPBA), and specifically the DOPS forms, from the ISCP [14, 16].

Introducing medical students to ex vivo model has been successful throughout a relatively extensive period of time [7, 8]. This remains a cost-effective option, given the fact that ex vivo modules are cheaper compared to when you attempt to include in vivo swine model, in order to achieve the highest fidelity model. Students seem to respond favourably to these initiatives, and their objective assessments show improvement in terms of the basic surgical skills [7].

In our case, we attempted to establish a combined course, which aims to help students consolidate the basic surgical knowledge, establish the principles of the surgical approach, and practice on the basic hands-on skills. The choice of modules (Table 2) was mainly based on the essence to cover from alpha to omega all the relevant experience that a medical student would need, in order to be safe and efficient in both theatre and surgical wards. The introduction of in vivo modules would be the ideal high-fidelity simulation environment, where medical students will be exposed to all theatre equipment and will perform their first manipulations with real tissue. This would build up students' confidence and safety, before they actually assist in theatre. The whole idea was to design a step-by-step approach, so that delegates would be able to take their skills forward to the next step. With regard to the in vivo transplant module, despite the limitations in the actual students' input, it still has a mentorship value as it is a unique opportunity to interact with the surgeon. Also the students are able to identify and consolidate step by step the thoracic anatomy as well as the cardiopulmonary physiology principles and understand the outlines of Transplant Surgery.

The question still remains about the cost effectiveness of the in vivo model in the undergraduate surgical education. Despite high cost being an obstacle, in vivo dissections were deemed really valuable in the feedback (Table 4). Furthermore, from the actual objective DOPS scores, we could assume that this was reflected in the mean difference in the scores before and after the in vivo modules (basic dissections and Heart and Transplant Surgery), which were statistically significantly improved (*P* < 0.005). Despite the limitations in the extent and detail of those assessments, we could support though that there is a benefit in the performance of the delegates.

MCQ exams, before and after completion of the course, were used to validate the quality of knowledge acquired from the basic science and case-based learning cores. The mean improvement was 2.33/32 (*P* < 0.005), and therefore this seems to work effectively. The MCQ exam is mainly designed to cover basic aspects of the taught lectures. In terms of the hands-on skills, we used DOPS forms to validate objectively the level of acquired competence. In every case, the scores were improved significantly after completion of each module (*P* < 0.005). For this purpose, we followed Miller's Pyramid Principles along with WPBA to validate it [16]. This follows the fundamental assumption that a young trainee (or a medical student in our case) would not be able to perform a skill completely independently; however, it still helps towards the acquisition of a higher level of competence on a specific skill on the scale 0–4. Finally, consolidation of knowledge is attempted via distribution of the international course manual, which aims to cover all the aspects of the course. We decided to distribute the manual on the participants' arrival, in order to achieve a similar background knowledge for every participant and hence to evaluate the effectiveness of the course itself. In any other case, it would have been more complicated to achieve a homogenous sample of students.

With regard to the DOPS scores, there has been an improvement in all modules before and after completion (*P* < 0.05). This was clear in both the objective comparison DOPS (Table 3), as well as in students' feedback (Table 4), suggesting that our course may be an effective teaching model. Also, the vast majority of scores suggest that students lack more in hands-on experience rather than anything else. There is significant improvement even in simple modules, like suturing.

Comparing British versus Greek students, we have to take into consideration that all British students are Year 3 students, whereas Greek students come from a mixture of Years 4–6. That may justify the slightly better performance of Greek students in the 2nd MCQ exam, as well as being more familiar with basic dissections (Skill 1), with no further significance. A more interesting comparison lies between the group consisting of Year 3 students in the UK and Year 4 students in Greece versus Years 5 and 6. Year 3 is the first clinical year in the UK, whilst Year 4 is the equivalent for Greek students. Again, on that occasion there are minor differences in the performance during the final MCQ exam as well as in in vivo module 1. This supports the gross homogeneity of the groups, as there are minor differences in the vast majority of the modules. Also, it underlines the lack of hands-on exposure, as final year students have similar performance with Year 3 and Year 4 students.

Finally, a last point worth commenting on is the selection of students and faculty from various institutions around Greece and the UK. There remains significant variation in surgical training amongst EU countries, despite all attempts to establish uniform qualifications, such as the European Boards. Therefore, it will be increasingly necessary to enhance the communication and interactions between medical students from diverse training systems to identify improvements and exchange ideas and views on training challenges. For that reason, it is very interesting to organize a joint course for British and Greek medical students.

From the medical students' point of view, they seemed to appreciate the acquired knowledge, which is directly reflected by their feedback (Table 4). The average overall feedback was 8.78/10 (7.72–9.87, SD = 0.56), and the students believe that this would be implemented on the Medical School Curriculum for the acquisition of the relevant competences (9.86/10, 8–10, and SD = 0.43). Also, they felt very strongly about having more confidence in approaching a surgical patient (9.50/10, 7–10, and SD = 0.86), which was improved from day 1 to day 2 (day 1 confidence 9.37/10, 7–10, and SD = 0.83). They also felt that they were well-supported throughout the course (9.03/10, 5–10, and SD = 1.44), which is also reflected in the good combination of faculty from the UK and Greece (9.10/10, 7–10, and SD = 1.06).

## 5. Conclusions

Wet lab simulation-based combined courses seem to be effective in the acquisition of theoretical knowledge, as well as hands-on skills for medical students. Combination of ex vivo and in vivo skills could potentially offer a step further in the preparation of the medical students, who would be interested to pursue a career in surgery. The question still remains as to what the right balance of time and money invested towards the most cost-effective model for this purpose is.

## Figures and Tables

**(a):** 

Domain	Rating N/D/S
(1) Describes indications, anatomy, procedure, and complications to assessor	
(2) Obtains consent, after explaining procedure and possible complications to patient	
(3) Prepares for procedure according to an agreed protocol	
(4) Administers effective analgesia or safe sedation (if no anaesthetist)	
(5) Demonstrates good asepsis and safe use of instruments and sharps	
(6) Performs the technical aspects in line with the guidance notes	
(7) Deals with any unexpected event or seeks help when appropriate	
(8) Completes required documentation (written or dictated)	
(9) Communicates clearly with patient and staff throughout the procedure	
(10) Demonstrates professional behaviour throughout the procedure	

**(b):** 

Level 0	Insufficient evidence observed to support a summary judgement
Level 1	Unable to perform the procedure under supervision
Level 2	Able to perform the procedure under supervision
Level 3	Able to perform the procedure with minimum supervision (needed occasional help)
Level 4	Competent to perform the procedure unsupervised (and could deal with any complications that arose)

**Table 2:** Delegates mean DOPS scores (N = 0/D = 1/S = 2) and global rating scale (0–4).

	Delegates mean DOPS scores (N = 0/D = 1/S = 2) and global rating scale (0–4)			
	Minimum	Maximum	Mean	Std. deviation
MCQ pre	9.00	22.00	15.3243	3.62900
MCQ post	9.00	25.00	18.2000	3.93016
In vivo dissections (N/D/S) pre	.00	1.00	.2308	.42967
In vivo dissections (N/D/S) post	1.00	2.00	1.2083	.41485
In vivo dissections Global Score (0–4) pre	1.00	2.00	1.2000	.41404
In vivo dissections Global Score (0–4) post	2.00	3.00	2.5000	.51887
Suturing Score pre (N/D/S)	.00	1.00	.8182	.39477
Suturing Score post (N/D/S)	1.00	2.00	1.9048	.30079
Suturing Global Rating pre	1.00	3.00	1.9375	.44253
Suturing Global Rating post	2.00	4.00	2.9375	.44253
IV Access Score pre (N/D/S)	.00	2.00	.7619	.53896
IV Access Score post (N/D/S)	1.00	2.00	1.6667	.48305
IV Access Global Rating pre	1.00	3.00	1.4286	.64621
IV Access Global Rating post	2.00	4.00	2.3571	.63332
ORIF Score pre (N/D/S)	.00	1.00	.8333	.38348
ORIF Score post (N/D/S)	1.00	2.00	1.8889	.32338
ORIF Global Rating pre	.00	3.00	1.9167	.66856
ORIF Global Rating post	2.00	4.00	3.0833	.51493
Wound Debridement pre (N/D/S)	.00	2.00	1.0588	.42875
Wound Debridement post (N/D/S)	2.00	2.00	2.0000	.00000
Wound Debridement Global Rating pre	.00	2.00	1.6154	.65044
Wound Debridement Global Rating post	2.00	3.00	2.8462	.37553
Heart and Transplant Global pre (0–4)	.00	1.00	.7500	.50000
Heart and Transplant Global post (0–4)	1.00	2.00	1.7500	.50000

**Table 3:** Comparison of the mean difference between MCQ (pre- and postcourse) and DOPS (pre- and postmodule).

Module	Comparison of MCQ and DOPS scores						
	Paired differences			95% confidence interval of the difference		*t*-test	Sig.
	Mean	SD	SE mean	Lower	Upper		
MCQ post-MCQ pre	2.66667	3.23715	.56352	1.51882	3.81451	4.732	.000
In vivo dissections (N/D/S)	.95833	.35864	.07321	.80689	1.10977	13.091	.000
In vivo dissections global (0–4)	1.28571	.61125	.16336	.93279	1.63864	7.870	.000
Suturing Score (N/D/S)	1.09524	.30079	.06564	.95832	1.23216	16.686	.000
Suturing Global (0–4)	1.00000	.51640	.12910	.72483	1.27517	7.746	.000
IV access (N/D/S)	.90476	.30079	.06564	.76784	1.04168	13.784	.000
IV access global (0–4)	.92857	.26726	.07143	.77426	1.08288	13.000	.000
ORIF (N/D/S)	1.05556	.41618	.09809	.84860	1.26252	10.761	.000
ORIF Global (0–4)	1.16667	.38925	.11237	.91935	1.41398	10.383	.000
Wound Debridement (N/D/S)	.93750	.44253	.11063	.70169	1.17331	8.474	.000
Wound Debridement Global (0–4)	1.23077	.59914	.16617	.86871	1.59283	7.407	.000
Heart and Transplant Global Score (0–4)	1.10000	.31623	.10000	.87378	1.32622	11.000	.000

**Table 4:** Overall feedback analysis (scale 0–10, 0 = disagree/worst, 10 = completely agree/best).

	Analysis of overall feedback (ascending values)						
	Range	Minimum	Maximum	Mean		Std. Deviation	Variance
	Statistic	Statistic	Statistic	Statistic	Std. error	Statistic	Statistic
ECG Workshop	7.00	3.00	10.00	**7.7187**	.29524	1.67012	2.789
Food	10.00	.00	10.00	**7.7333**	.42327	2.31834	5.375
Surgical Oncology (IL)	4.00	5.00	9.00	**7.7667**	.27411	1.50134	2.254
Website	6.00	4.00	10.00	**7.7931**	.33432	1.80038	3.241
Breaks	10.00	.00	10.00	**7.8000**	.51950	2.84544	8.097
Rectal Cancer (IL)	8.00	2.00	10.00	**7.8750**	.47895	2.34637	5.505
ENT CbL	7.00	3.00	10.00	**7.9355**	.34992	1.94826	3.796
Heart and Transplant In Vivo Module (Sk6)	7.00	3.00	10.00	**7.9667**	.39966	2.18905	4.792
Vascular CbL	6.00	4.00	10.00	**7.9677**	.32622	1.81629	3.299
Interest in Surgical Career	7.00	3.00	10.00	**8.1875**	.34907	1.97464	3.899
Cardiothoracic CbL	8.00	2.00	10.00	**8.2813**	.30531	1.72710	2.983
Fluids and Analgesia Workshop	6.00	4.00	10.00	**8.4687**	.27307	1.54470	2.386
Basic Science Workshops Overall	6.00	4.00	10.00	**8.6774**	.32918	1.83280	3.359
DKA/ACS/Sepsis CbL	4.00	6.00	10.00	**8.7097**	.16844	.93785	.880
Research in Medical Education (IN)	5.00	5.00	10.00	**8.7187**	.26272	1.48616	2.209
In vivo dissections (Sk1)	5.00	5.00	10.00	**8.7500**	.24593	1.39122	1.935
Orthopaedics CbL	7.00	3.00	10.00	**8.7742**	.32696	1.82043	3.314
Statistic Mean Feedback	**2.15**	**7.72**	**9.87**	**8.7882**	**.08485**	**.56284**	**.317**
Advances in Valve Surgery (IL)	5.00	5.00	10.00	**8.8333**	.29588	1.62063	2.626
GS and HPB CbL	4.00	6.00	10.00	**8.8437**	.18022	1.01947	1.039
Wound Debridement – Ex Vivo (Sk5)	6.00	4.00	10.00	**8.8710**	.26938	1.49982	2.249
Abdominal Trauma CbL	5.00	5.00	10.00	**8.8710**	.26535	1.47743	2.183
Consent CbL	7.00	3.00	10.00	**8.8710**	.23981	1.33521	1.783
Burns CbL	5.00	5.00	10.00	**8.9063**	.23053	1.30407	1.701
IV Access – Ex Vivo (Sk3)	3.00	7.00	10.00	**8.9375**	.19540	1.10534	1.222
Shock Workshop	3.00	7.00	10.00	**8.9375**	.19017	1.07576	1.157
Suturing Ex Vivo (Sk2)	7.00	3.00	10.00	**8.9687**	.27121	1.53422	2.354
Chapters Selection in Manual	3.00	7.00	10.00	**9.0000**	.15554	.87988	.774
Appearance of the Manual	4.00	6.00	10.00	**9.0000**	.17961	1.01600	1.032
ESMSC “safety triangle”	5.00	5.00	10.00	**9.0000**	.23187	1.29099	1.667
Lecture Surgical Approach (IN)	4.00	6.00	10.00	**9.0000**	.20080	1.13592	1.290
Support during Course	5.00	5.00	10.00	**9.0333**	.26472	1.44993	2.102
Was it a useful course (Day 1)	3.00	7.00	10.00	**9.0625**	.17925	1.01401	1.028
Faculty Selection	3.00	7.00	10.00	**9.1000**	.19387	1.06188	1.128
ORIF- Ex Vivo	7.00	3.00	10.00	**9.1290**	.27334	1.52188	2.316
Course Lead	9.00	1.00	10.00	**9.1724**	.32570	1.75395	3.076
ABG Workshop	4.00	6.00	10.00	**9.2813**	.18640	1.05446	1.112
Was it a useful course (Day 2)	4.00	6.00	10.00	**9.3000**	.18036	.98786	.976
General Concept Day 1	5.00	5.00	10.00	**9.3000**	.20982	1.14921	1.321
General Concept Day 2	3.00	7.00	10.00	**9.3438**	.14625	.82733	.684
Hand Emergencies CbL	3.00	7.00	10.00	**9.3548**	.16427	.91464	.837
Surgical Approach Ability – Day 1	3.00	7.00	10.00	**9.3750**	.14722	.83280	.694
Overall Rate of the Idea	2.00	8.00	10.00	**9.4194**	.12930	.71992	.518
Facilities	3.00	7.00	10.00	**9.4667**	.14169	.77608	.602
Surgical Approach Ability Day 2	3.00	7.00	10.00	**9.5000**	.15720	.86103	.741
Should Medical School Provide It? (Feedback from Day 1)	3.00	7.00	10.00	**9.7813**	.10752	.60824	.370
Should Medical School Provide It? (Feedback from Day 2)	2.00	8.00	10.00	**9.8667**	.07927	.43417	.189

CbL = case-based lecture; IL = invited lecture; IN = introductory lecture; Sk = in vivo/ex vivo modules.
